# Influence of Murine Mesenchymal Stem Cells on Proliferation, Phenotype, Vitality, and Cytotoxicity of Murine Cytokine-Induced Killer Cells in Coculture

**DOI:** 10.1371/journal.pone.0088115

**Published:** 2014-02-06

**Authors:** Martin Bach, Christoph Schimmelpfennig, Alexandra Stolzing

**Affiliations:** 1 Fraunhofer Institute for Cell Therapy and Immunology, Leipzig, Germany; 2 Universitätsklinik Leipzig, Department für Innere Medizin, Neurologie und Dermatologie, Sektion Rheumatologie, Leipzig, Germany; 3 Klinikum St. Georg, Klinik für Internistische Onkologie und Hämatologie, Leipzig, Germany; University of Pittsburgh, United States of America

## Abstract

Stimulating lymphocytes with Ifn-γ, anti-CD3, and interleukin-2 promotes the proliferation of a cell population coexpressing T-lymphocyte surface antigens such as CD3, CD8a, and CD25 as well as natural killer cell markers such as NK1.1, CD49, and CD69. These cells, referred to as cytokine-induced killer cells (CIKs), display cytotoxic activity against tumour cells, even without prior antigen presentation, and offer a new cell-based approach to the treatment of malignant diseases. Because CIKs are limited in vivo, strategies to optimize in vitro culture yield are required. In the last 10 years, mesenchymal stem cells (MSCs) have gathered considerable attention. Aside from their uses in tissue engineering and as support in haematopoietic stem cell transplantations, MSCs show notable immunomodulatory characteristics, providing further possibilities for therapeutic applications. In this study, we investigated the influence of murine MSCs on proliferation, phenotype, vitality, and cytotoxicity of murine CIKs in a coculture system. We found that CIKs in coculture proliferated within 7 days, with an average growth factor of 18.84, whereas controls grew with an average factor of 3.7 in the same period. Furthermore, higher vitality was noted in cocultured CIKs than in controls. Cell phenotype was unaffected by coculture with MSCs and, notably, coculture did not impact cytotoxicity against the tumour cells analysed. The findings suggest that cell–cell contact is primarily responsible for these effects. Humoral interactions play only a minor role. Furthermore, no phenotypical MSCs were detected after coculture for 4 h, suggesting the occurrence of immune reactions between CIKs and MSCs. Further investigations with DiD-labelled MSCs revealed that the observed disappearance of MSCs appears not to be due to differentiation processes.

## Introduction

Stimulating lymphocytes with interferon-γ (Ifn-γ), anti-CD3, and interleukin (IL)-2 leads to the selection and proliferation of cells expressing T-lymphocyte surface antigens such as CD3, CD8a, and CD25 as well as natural killer (NK) cell markers such as NK1.1, CD49, and CD69 [1]–[3]. These cells, referred to as cytokine-induced killer cells (CIKs), mediate major histocompatibility complex-unrestricted cytotoxic activity against target cells even without prior antigen presentation [1]. Several studies have attested to the potency of CIKs in lysing tumour cells [4]–[6], and CIKs are promising new options in the treatment of malignant diseases.

Peripheral blood lymphocytes contain only ∼5% CIKs [3]. For efficient treatment, CIKs must therefore be expanded in vitro before transplantation back into patients. Many efforts have been made to optimize the yield of in vitro CIK enrichment. One approach is to use alternative cytokines for stimulation, such as IL-7 or IL-12 instead of IL-2. The replacement of IL-2 by IL-12 improves cytotoxicity, but simultaneously lowers proliferation rates. The use of IL-7 has no distinct advantages [2], [7]. Use of bispecific antibodies, such as anti-CD3/anti-CA125 or anti-CD3/anti-Her2, has been found to induce CIK-mediated lysis of otherwise CIK-resistant ovarian carcinoma cells; however, this approach does not yield increased proliferation rates [8]. Another study reported that the anti-tumour activity of CIKs can be improved through transfection with oncolytic viruses [9] or genes for tumour-specific receptors [10]. Cocultures of CIKs with dendritic cells have yielded increased CIK proliferation and cytotoxicity, as well [11]. Even higher cytotoxicities are observed when idiotype-pulsed dendritic cells are used [12]. Against this background, the present study investigated the interactions between CIKs and mesenchymal stem cells (MSCs) in a coculture system.

MSCs are multipotent adult stem cells that physiologically reside in tissues such as bone marrow [13], adipose tissue [14], amniotic fluid [15], connective tissue [16], and many others [17]–[20]. Owing to varying stem cell niches, MSCs are a heterogeneous cell population in terms of differentiation potential, proliferation capacity, phenotype, and other characteristics [21], [22]. Aside from the niche conditions, various isolation and cultivation protocols, donor sex and age, choice of media, and especially species-related distinctions contribute to the remarkable heterogeneity of MSCs [21]. This heterogeneity has led to a considerably incomplete understanding of MSCs what is reflected in an inconsistent nomenclature [21] and in partially contradictory characterizations of MSCs. The International Society for Cell Therapy (ISCT) has therefore proposed criteria for characterization of human MSCs, including adherence to plastic surfaces, the capability to differentiate into osteoblasts, adipocytes, and chondrocytes, and phenotypical characters [28]. The identification by phenotyping is not trivial. Indeed, a variety of phenotypical characteristics appears in the ISCT criteria and the literature; however, none of these markers is unique for MSCs. Aside from preanalytical challenges and species-related distinctions, the difficulty in identification is certainly due to the aforementioned heterogeneity of MSCs. Therefore, a combination of positive and negative markers should be used to characterize MSCs.

Working with MSCs from different species complicates identification further. Although many of the criteria proposed for human MSCs are also applicable to the murine system, substantial distinctions among MSCs of different species have been reported [31], [50]. Therefore, the murine MSCs used in this study were characterized under consideration of the ISCT criteria and additionally under consideration of publications dealing with murine MSCs. The following criteria were considered: adherence to plastic surfaces [25], [26], [27]; spindle-shaped, fibroblast-like morphology [27], [54]; growth in the form of colony-forming units (CFUs) and capability of differentiation into osteoblasts [21]; and phenotypical features [15], [28], [44]–[46], [51]–[53]. Many previous publications using the identical protocol for MSC isolation have also shown adipocyte and chondrocyte differentiation [13].

MSCs show many immunomodulatory characteristics, which makes them a promising tool for use in therapies. For example, human MSCs display MHC-unrelated suppression of T-lymphocyte proliferation; this effect has been observed on both naive as well as mature T-lymphocytes. Thereby, T-lymphocytes do not become apoptotic or anergic, as they are stimulable after removal from the MSC milieu [31]. The proliferation of NK cells [32], B cells [33], and dendritic cells [30] is also reportedly suppressed by MSCs.

The immunomodulatory effects of MSCs are multifaceted and, to some extent, even contrary. Many reports have highlighted the immunosuppressive characteristics of MSCs. Götherström [30], for example, reported the case of a 9-year-old boy who developed acute graft-versus-host disease (GvHD) IV after receiving haematopoietic stem cell transplant from a matched unrelated donor for treatment of leukaemia and who was resistant to established forms of immunosuppression. Infusions of haploidentical MSCs derived from the patient’s mother reversed the GvHD. Eight additional patients with steroid refractory GvHD III–IV were treated with MSC infusions and showed no side effects. Six of the eight patients displayed complete response.

In contrast, other groups have reported that MSCs do not show constitutional immunosuppressive or antiproliferative effects [31]–[36]. These studies postulate a more differentiated point of view that requires consideration of a multitude of conditions and external influences, such as the stage of activation of cocultured lymphocytes/NK cells or the ratio between MSCs and lymphocytes/NK cells. In this study, we investigated the influence of murine MSCs on proliferation, phenotype, vitality, and cytotoxicity of murine CIKs in a coculture system.

## Materials and Methods

### Ethics Statement

Mice used for isolation of splenocytes and bone marrow cells were raised and housed in the animal facilities of the Faculty of Medicine, University of Leipzig, according to European (Council Directive 86/609/EEC) and German (Tierschutzgesetz) guidelines for the welfare of experimental animals. The killing of animals was presented to and approved by local authorities (Landesdirektion Sachsen, TV T13/09).

### Isolation and Cultivation of MSCs and DiD Staining

8- to 12-week-old C57/black 6 mice were killed using CO2, and their femora and tibiae were removed and cleaned of skin and muscle. According to the method of Dobson [25], the proximal and distal ends of the murine femora and tibiae were removed, the bones were inserted into Eppendorf tubes, and bone marrow was obtained after centrifugation (10 min, 250×g). Bone marrow cells were then collected in medium (RPMI 1640, 10% foetal calf serum [FCS], 1% 1 M HEPES, 0.5% gentamycin, and 0.1% mercaptoethanol; all purchased from Gibco, Invitrogen, Germany). To obtain adherent cells, 5.4×107 cells per T25 flask (Greiner, BIO, Germany) were plated and cultured at 5% CO2 and 37°C. After 72 h, the supernatants, and therefore all suspended cells, were discarded, and the medium was replaced. On day 7 (cells nearly reached confluence), the cells were either used for coculture with CIKs or were harvested for flow cytometry.

The medium in some of the flasks was removed after 6 days and replaced with 3.5 ml of fresh medium supplemented with DiD staining solution (5 µl DiD/ml; Vybrant staining solution, Invitrogen). After incubation with the staining solution for 30 min, the supernatant was discarded, and the cells were washed with phosphate-buffered saline (PBS). Finally, fresh medium was added and DiD-labelled cells were placed in the incubator until use the following day.

### Isolation and Cultivation of Control CIKs

8- to 12-week-old C57/black 6 mice were killed using CO2, and their spleens were removed and pressed through a filter (pore size, 100 µm; Greiner BIO One) into Petri dishes containing PBS (supplemented with 1% FCS and 0.5% gentamycin [Gibco, Invitrogen]). Cells were washed with PBS, incubated with erythrocyte lysis buffer (Gibco, Invitrogen) for 1 min, and washed again before medium was added (RPMI 1640, 10% FCS, 1% 1 M HEPES, 0.5% gentamycin, and 0.1% mercaptoethanol [Gibco, Invitrogen]). Cells were counted using trypan blue and plated at a concentration of 2×106 cells per millilitre of culture in T75 cell culture flasks (Greiner, BIO). Recombinant murine (rm)-Ifn-γ (1.000 U/ml; Immunotools, Germany) was added, and cells were incubated (5% CO2 and 37°C). After 1 day, 50 ng/ml anti-CD3 antibodies (OKT3, BD-Pharma) and 300 U/ml rm-IL-2 (Immunotools) were added. Cells were passaged and counted on days 3, 7, 11, 14, and 17, and medium as well as rm-IL-2 were replaced in appropriate amounts.

### CIK/MSC Cocultures

On day 7, supernatants from the nearly confluent MSC flasks (containing 0.8×106 MSCs) were discarded, and 3.6 million 7-day-old allogeneic CIKs, fresh medium, and appropriate amounts of rm-IL-2 were added. On days 11 and 14, two-thirds or half of the medium, and therefore that number of suspended cells, were removed and replaced with fresh medium and an adequate amount of rm-IL-2 (300 U/ml) such that cocultured CIKs (cCIKs) were split at a ratio 1∶3 or 1∶2, respectively.

### CIKs in MSC-conditioned Medium

MSC supernatants were removed on day 7 and centrifuged to ensure that no cells were left in the supernatant media. Then, two-thirds of the cell suspension from 7-day-old CIK cultures, and thus two-thirds of the CIKs were withdrawn and replaced with MSC-conditioned medium such that CIKs were split at a ratio of 1∶3. Rm-IL-2 was substituted in the appropriate amount (300 U/ml conditioned medium). The same procedure was carried out on days 11 and 14.

### Cultivation of Target Cells

Cells of an adherent fibrosarcoma cell lineage (Wehi 164 S; DSMZ, Braunschweig, Germany) were plated (RPMI medium 1640, 10% FCS, and 1% penicillin/streptomycin, purchased from Gibco, Invitrogen) at a density of 1.5 million cells/75 cm^2^ and passaged every 2–3 days.

### Flow Cytometry

CIKs and MSCs were harvested, transferred into PBS, and incubated with Fc-solution (Beckman Coulter) for 5 min at 4°C to prevent unspecific antibody binding. During the incubation with Fc-blocking reagent, antibodies were placed in fluorescence-activated cell sorter (FACS) tubes (Greiner, BIO). The following antibodies, purchased from Beckman Coulter (final concentrations in brackets), were used: CD3-FITC (1∶100), CD4-PE (1∶250), CD8a-PE (1∶250), CD11b-PE (1∶160), CD19-PE (1∶250), CD25-PE (1∶250), CD45-PE (1∶250), CD49-PE (1∶250), NK1.1-PE (1∶60), CD69-PE (1∶250), HamIgG-FITC-isotype-control (1∶250), and RatIgG2a-PE-isotype-controll (1∶250). In addition, CD34-PE (Caltaq, 1∶100), CD44-AF488 (Biozol, 1∶250), CD73-PE (Biozol, 1∶100), CD90 (Thy1.2)-FITC (Miltenyi, 1∶100), and CD166 (Antikörper-online, 1∶100).

After incubation for 5 min, Fc-blocked cells were added to the antibody-containing FACS tubes, incubated for 20 min at 4°C, and washed with PBS. Then, 7-aminoactinomycin-D (7-AAD; 1∶100, Beckman Coulter) stain was added, and the cells were incubated for 5 min at 4°C. Either isotypes or autofluorescence served as controls. Cells were gated on vital cells, indicated by low 7-AAD signals.

### Cytotoxicity Assay

A lactate dehydrogenase (LDH)-releasing assay (Roche) was performed according to the manufacturer’s protocol to assess the influence of MSCs on CIK cytotoxicity. Fourteen-day-old cCIKs and control CIKs were incubated with target cells (Wehi 164 S, fibrosarcoma cell lineage, DSMZ) in 96-well plates at various effector cell:target cell ratios (1∶1 to 50∶1). After 4 h, the plates were centrifuged, and 100 µl of supernatants were transferred to fresh 96-well plates. Subsequently, an LDH-substrate mixture (100 µl, content of the kit) containing tetrazolium salt INT was added to the supernatants. After incubation for 30 min in dark at room temperature, absorbance was determined at a wavelength of 492 nm against a reference wavelength of 600 nm. Lysis was calculated using the following equation:
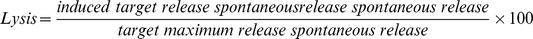



### CFU-F-Assay

In a modified version of the technique originally described by Kuznetsov et al. [37], 10 million bone marrow cells were suspended in either 5 ml of normal medium (DMEM low glucose, 10% FCS, and 1% penicillin/streptomycin; all purchased from Gibco, Invitrogen) or osteogenic medium (normal medium, supplemented with dexamethasone [1 µl/ml] and ascorbic acid [50 µg/ml]; purchased from AppliChem, Germany). The medium was first changed after 72 h and then after every 3–4 days. On day 14, cells were washed with PBS and fixed in ethanol at 4°C for 20 min. Fixed cells were stained with alkaline phosphatase (ALP) staining solution (Tris, dimethylformamide, naphthol ASBI, and fast red; all purchased from AppliChem) and photographed. Cells were then discoloured by incubation with 90% ethanol overnight, stained with methylene blue (AppliChem), and then were photographed again.

### Statistics

The Mann-Whitney U-test was used to assess statistical significance. Differences with a p value of <0.05 were considered statistically significant. Error indicators reflect SEM (standard error of the mean).

## Results

### CIK Proliferation Effects of Coculturing CIKs with MSCs and Cultivating CIKs in MSC-conditioned Medium

Many strategies for optimizing the culture conditions of CIKs have been investigated. In this study, the effects of an MSC/CIK coculture system on CIKs were examined. Seven-day-old CIKs were cocultured with MSCs for 10 further days (cCIKs).

To investigate the influence of humoral factors, some of the CIKs were cultured in cell-free MSC supernatants from day 7 onward (mediaCIKs). The influences of potential immune reactions of CIKs against MSCs were excluded by centrifuging the supernatants before addition to CIKs.

**Figure 1:**
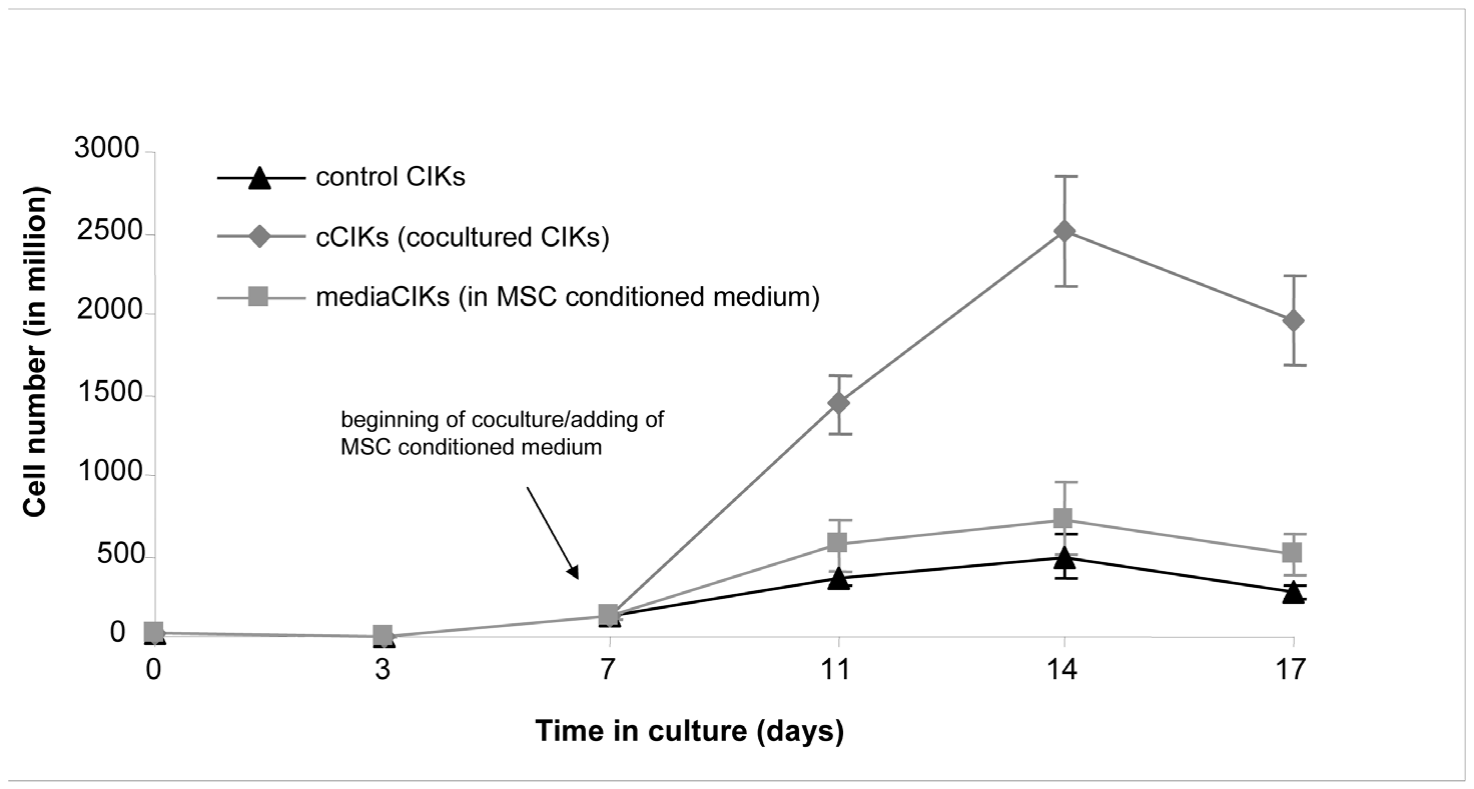
Comparison of cytokine-induced killer cell (CIK) proliferation. CIKs were cultivated according to protocol (controls). On day 7, some of the CIKs were cultivated either with mesenchymal stem cells (cCIKs) or in MSC-conditioned medium (mediaCIKs), and proliferation was compared with that of controls. After starting the coculture, cCIKs proliferated faster than controls (p<0.05). The mediaCIKs displayed increased proliferation (p>0.1), too, but proliferation rates did not reach the level of cCIK proliferation.

Cell numbers were determined using the trypan blue method on days 0, 3, 7, 11, 14, and 17. After day 7 (beginning of coculture), the number of cCIKs increased from 133 million to 2,515 million (day 14) and that of mediaCIKs increased from 133 million to 733 million. In the same period, the number of control cells increased from 133 million to 495 million. After day 14, the absolute cell numbers of controls, cCIKs, and mediaCIKs decreased (Figure 1).

### Effects on Phenotype

**Figure 2:**
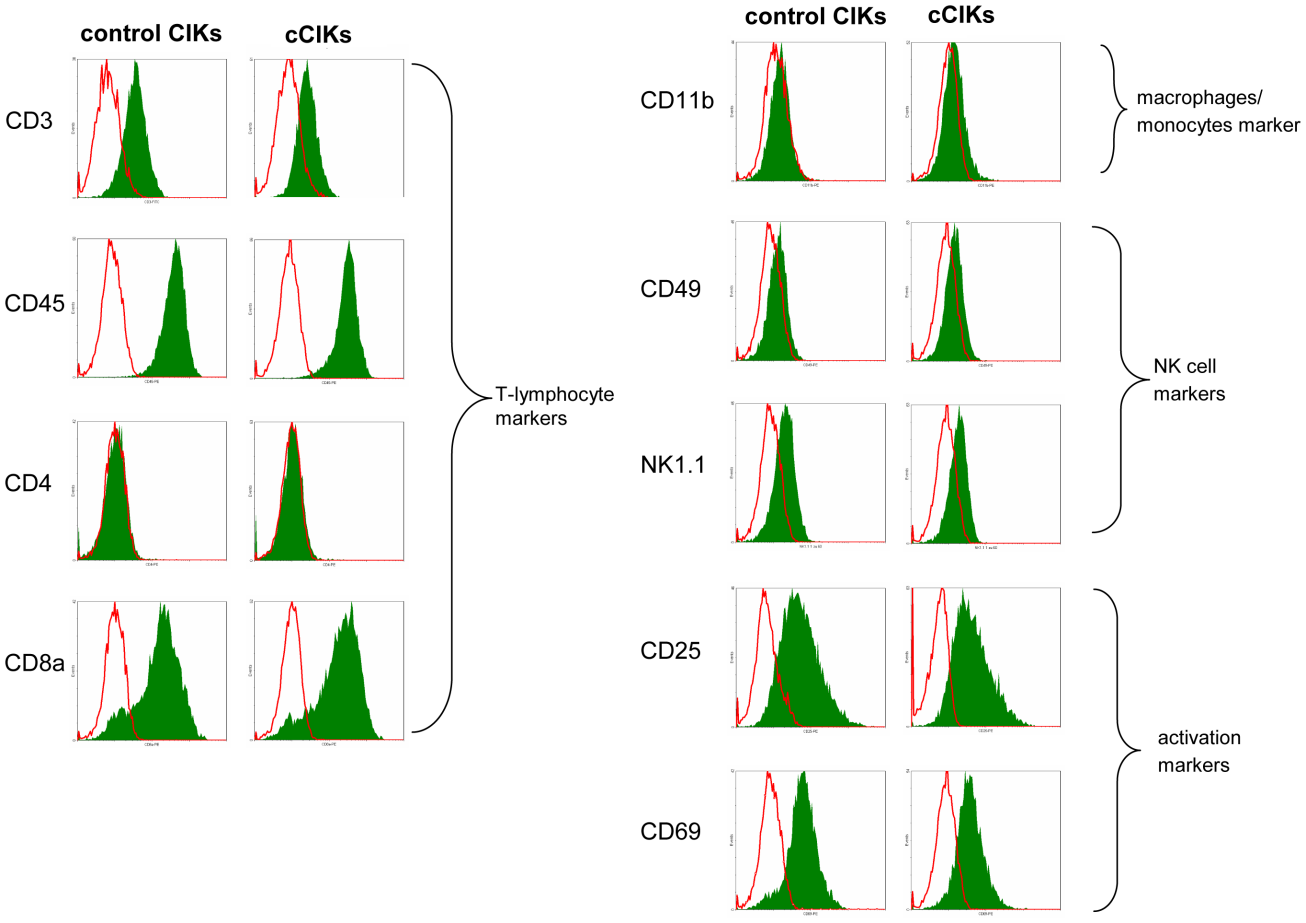
Comparison of CIK phenotypes. Fluorescence-activated cell sorter (FACS) analyses of control CIKs and cCIKs were performed on day 14. Controls expressed typical CIK markers, including T-cell markers CD3, CD8a, and CD25 and natural killer (NK) cell markers such as CD49, CD69, and NK1.1. No differences were observed between control CIKs and cCIKs.

For investigating the effects of coculture on CIK phenotype, controls and cCIKs were harvested on day 14 (after 7 days of coculture) and analysed using flow cytometry. The results demonstrated that the phenotypes of controls and cCIKs were identical (Figure 2). All cells were positive for CD3 and CD45. CD4 was absent, whereas CD8a was highly expressed on both cCIKs and controls. Only low signals of CD11b were detectable. The signals from NK cell markers CD49 and NK1.1 were weak for controls as well as for cCIKs. Strong CD25 and CD69 signals reflected an advanced stage of cell activation.

### Effects on CIK Vitality

**Figure 3:**
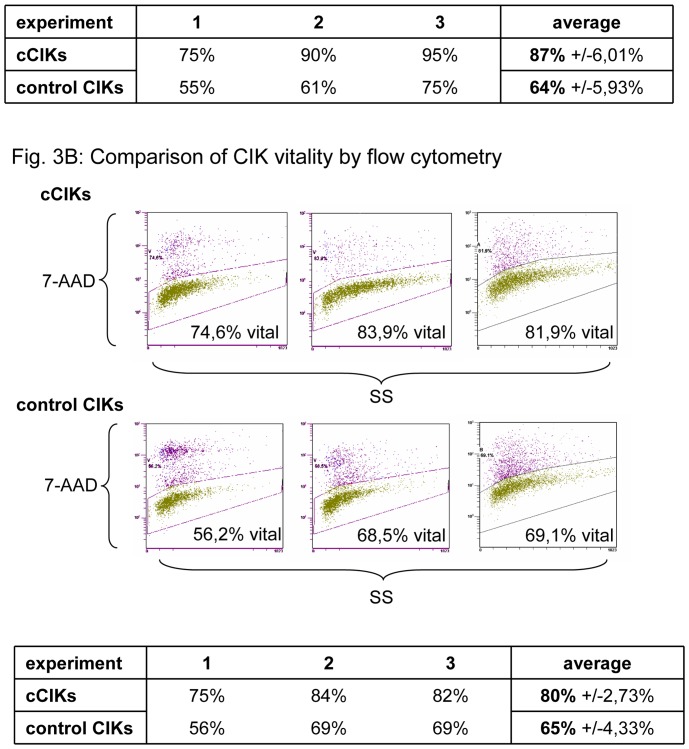
Comparison of CIK vitality. Vitalities of control CIKs and cCIKs were assessed on day 14 using two different methods. Part A displays vitalities determined using trypan blue staining. No significant differences in CIK viability were found at the 0.05 level (p = 0.1). Part B shows comparisons of CIK vitality, as assessed using 7-AAD staining. No significant differences were found (p = 0.1).

The vitalities of three different cultures from day 14 were assessed by trypan blue staining and via flow cytometry (7-AAD staining). Figure 3A presents the vitalities determined using trypan blue staining. The vitality of cCIKs was 87% and that of controls was 64% of total cells (p = 0.1). Figure 3B shows the related dot plots of 7-AAD staining from flow cytometry. Vital cells have low signal intensities, whereas the staining solution passes through the damaged membranes of non-vital cells, as reflected by high signal intensities. 80% of cCIKs showed low signal intensities suggestive of vital cells. Low intensities were seen in 65% of controls, but the difference was not significant (p = 0.1).

### Effects on CIK Cytotoxicity

**Figure 4:**
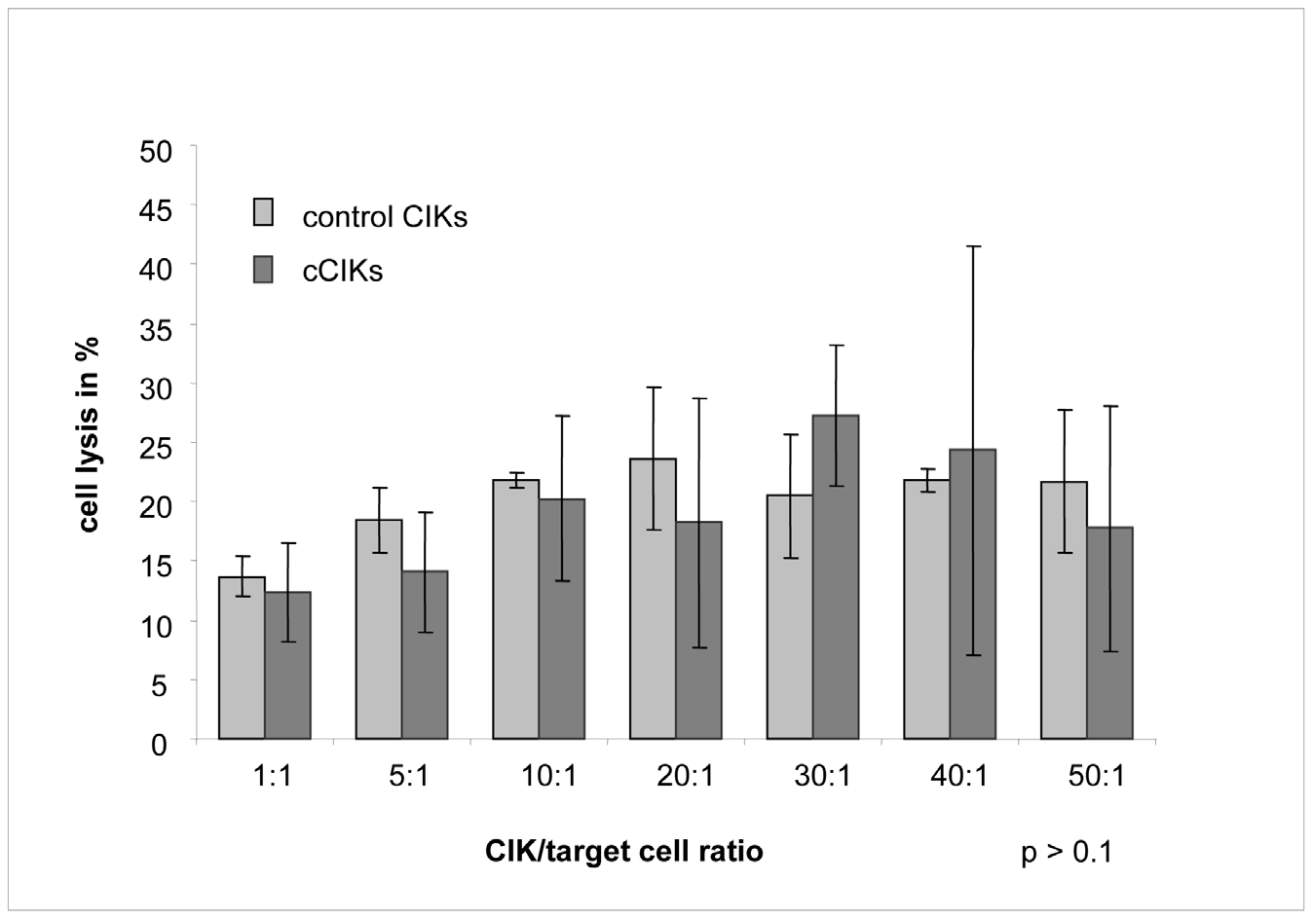
Comparison of CIK lysis potential against tumour cells. Lysis rates of control CIKs compared with those of cCIKs against fibrosarcoma cells (Wehi 164 S) on day 14 for different effector-cell/target-cell ratios. Error indicator represents standard error of the mean. Initially, lysis rates increased with increasing effector-cell/target-cell ratio up to a lysis rate of 27% at a ratio of approximately 30∶1. Further increases in the ratios did not increase lysis rates further. No significant differences were observed between control CIKs and cCIKs (p>0.1).

Cytotoxicity is a crucial variable of CIKs in anti-tumour therapy applications. An LDH-releasing assay was performed to determine the cytotoxicity of CIKs against tumour cells (Wehi 164 S). Figure 4 shows the average lysis rates of controls and cCIKs for various effector-cell/target-cell ratios. Initially, lysis rates increased with increasing effector-cell/target-cell ratio, up to a lysis rate of 27% at a ratio of approximately 30∶1. Further increases in the ratios did not increase lysis rates further. No significant differences were found between controls and cCIKs at any of the tested ratios (p>0.1).

### MSC Characterization

**Figure 5:**
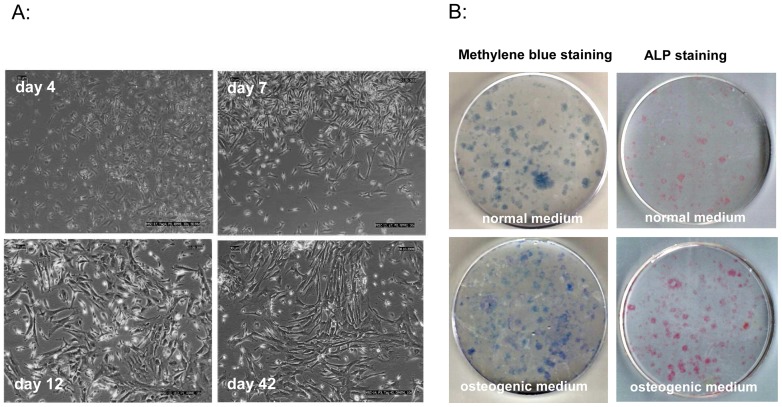
MSC characterization. A: MSCs were evaluated under a light microscope and representative photographs were captured on days 4, 7, 12, and 42 of incubation. Cells show typical fibroblast morphology and undergo typical time-dependent transitions among three morphologically distinct cell types: thin spindle-shaped cells, wider spindle-shaped cells, and still wider spindle-shaped cells. B: Fibroblastic colony-forming unit assays (CFU-F) were performed for 14 days. Colonies were then stained for ALP and methylene blue. When cultivated in normal medium, only a few colonies expressed ALP (line 1), whereas nearly all of the colonies stained with methylene blue also showed ALP expression when cultivated in osteogenic medium (line 2).

The MSCs used in this study were characterized by their adherence to plastic surfaces; by their spindle-shaped, fibroblast-like morphology; by their capability to differentiate in CFU-F-Assays into osteoblasts; and by phenotypical features. To illustrate the spindle-shaped, fibroblast-like morphology, we evaluated MSCs with light microscopy (10× augmentations), and representative photographs were taken on days 4, 7, 12, and 42. As is characteristic for MSCs, the cells underwent time-dependent transitions from thin spindle-shaped cells to wider spindle-shaped cells to still wider spindle-shaped cells (Figure 5A).

The potential to proliferate in CFUs and differentiate in the presence of dexamethasone and ascorbic acid into osteoblasts is shown in Figure 5B, which shows adherent bone marrow cells growing in colonies. When cultivated in normal medium (without dexamethasone and ascorbic acid) only a few of the total colonies that could be observed with nonspecific methylene blue staining expressed alkaline phosphatase (ALP). In contrast, when cells were cultivated in osteogenic medium (containing dexamethasone and ascorbic acid), most of the colonies presented in total colony staining were simultaneously positive for ALP.

**Figure 6:**
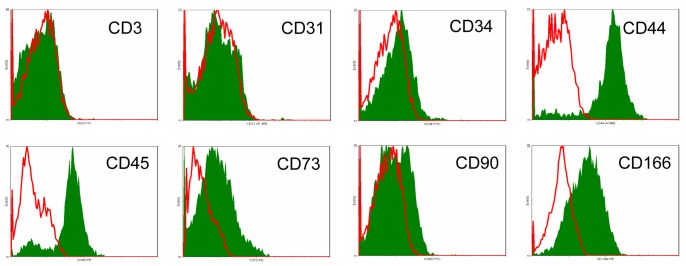
MSC phenotyping. MSCs were harvested and analysed with flow cytometry for a panel of MSC-characterizing markers on day 7. Thus, the presented phenotype resembles that of MSCs used in the cocultures. MSCs were negative for CD3, CD31, CD34, and CD90. Positive signals were measured for CD44, CD73, and CD166. Positive as well as negative signals were detected for CD45.

For phenotypical characterization, some of the MSCs were harvested on day 7 and analysed using flow cytometry. Because the cocultures started on day 7, the phenotype shown in Figure 6 reflects the phenotype of the MSCs used for the cocultures. MSCs were negative for CD3, CD31, CD34, and CD90. Positive signals were detected for CD44, CD73, and CD166, and positive as well as negative signals were detected for CD45.

### Phenotype of Adherent Cells after Coculture

**Figure 7:**
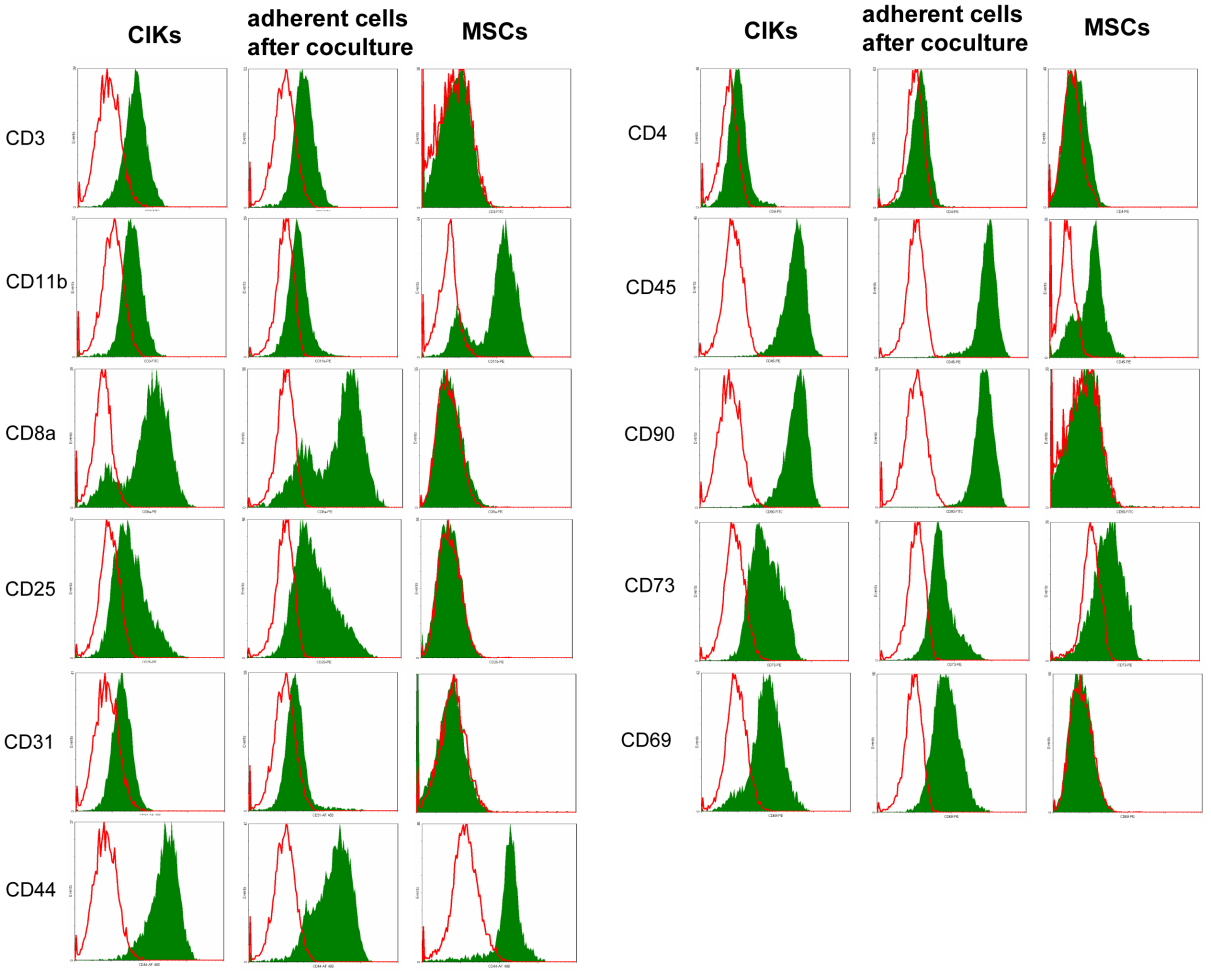
Comparison phenotypes: CIKs – adherent cells after coculture - MSCs. After 7/MSC coculture (day 14), adherent cells were harvested separately from suspended cells and analysed using flow cytometry. In addition, 14-day-old MSCs and CIKs were harvested and analysed for the same markers. Adherent cells after coculture (column in the middle) display the phenotype of CIKs (left column) and differ considerably from the phenotype expected for MSCs (right column).

When adherent cells were evaluated under light microscope at the end of coculture, they were found to have lost their typical spindle-shaped morphology. Surprisingly, the cells looked like CIKs instead (data not shown). To identify the adherent cells in the coculture flasks, we harvested these cells at the end of the coculture and analysed them using flow cytometry for MSC- and CIK-specific surface markers. The cells were found to express the surface markers for CIKs, not MSCs (Figure 7).

### DiD-labelled MSCs

As shown in Figure 7, adherent cells after coculture displayed the same phenotypical markers as CIKs and no longer resembled MSCs. To investigate whether MSCs in coculture had been lysed or had changed their phenotype via differentiation or cell fusion, we performed coculture experiments using DiD-labelled MSCs.

**Figure 8:**
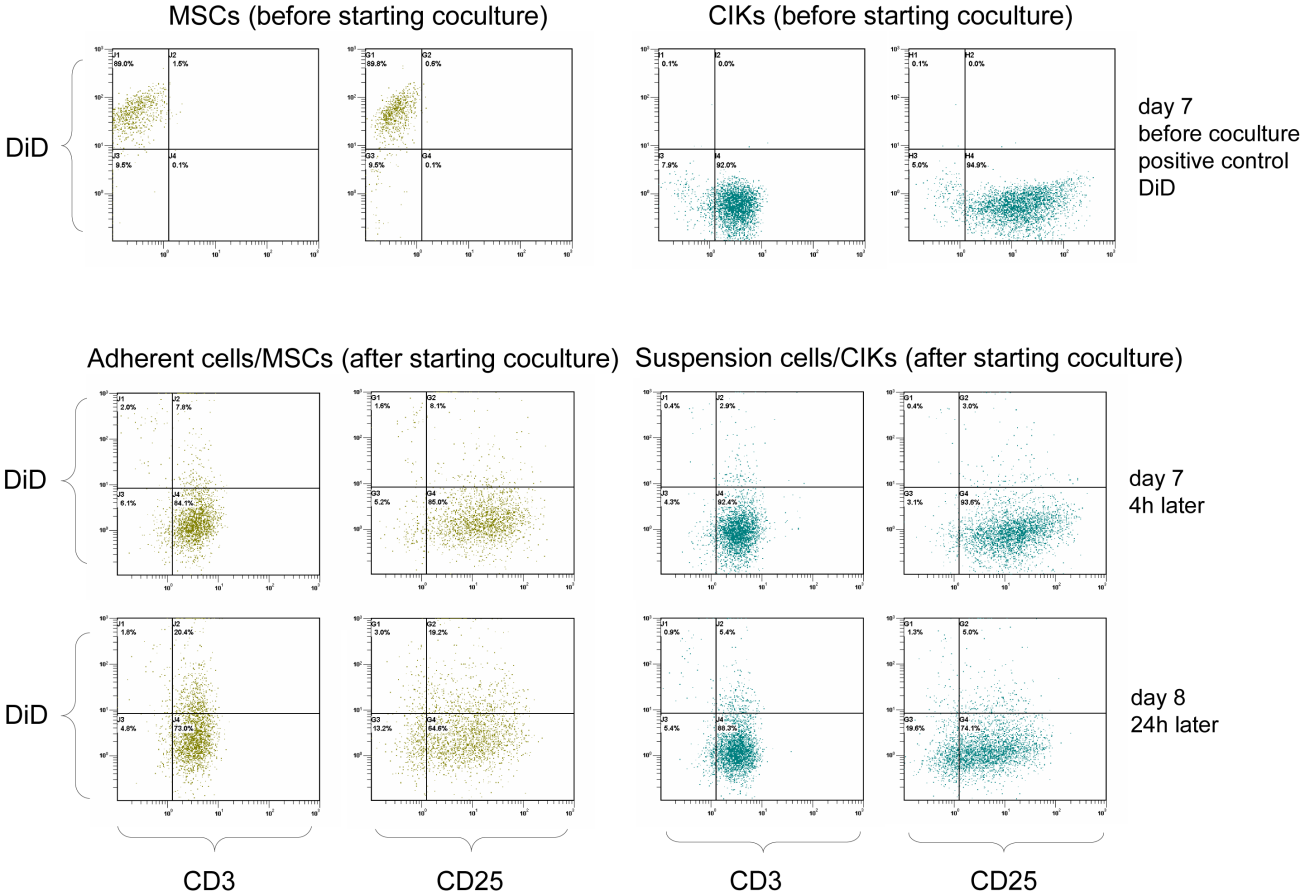
DiD-labelled MSCs. To investigate whether cocultured MSCs were differentiated, we carried out coculture experiments using DiD-labelled MSCs. Suspended cells (cCIKs) and adherent cells were harvested separately from each other after 4 and 24 h of coculture, stained with anti-CD3 and anti-CD25, and analysed using flow cytometry. The first line shows the results of flow cytometry analysis just before the addition of CIKs to the labelled MSCs. Clearly positive DiD signals were seen at the MSCs (positive control). However, CD3 and CD25 were negative, which was expected for MSCs. The CIKs were not labelled and were therefore DiD negative. As for CIKs characteristic, CD3 and CD25 were positive. The second line shows the results of FACS analysis of adherent cells and suspended cells after 4 h of coculture. Adherent cells have switched phenotype from CD3−/CD25− to CD3+/CD25+. DiD signals of adherent cells have disappeared. Suspended cells were still CD3+/CD25+ and DiD−. The third line (after 24 h) confirms the results seen after 4 h. Adherent cells showed the phenotype of the suspended cells and were DiD−.

Some DiD-labelled MSCs were harvested and analysed before starting the coculture (Figure 8, first line). A clearly positive signal for DiD was measured, indicating successful DiD labelling the previous day (positive control). Corresponding to the MSC phenotypes presented in Figure 6 and Figure 7, these cells too were CD3− and CD25−. In contrast, CIKs were CD3+ and CD25+ and DiD negative (because they were unlabelled).

After 4 h of coculture, adherent cells and suspended cells were harvested separately, stained for CD3/CD25, and re-analysed using flow cytometry (see Figure 8, second line). In contrast to the previous analysis, adherent cells switched from CD3−/CD25− to CD3+/CD25+, and the DiD signal disappeared. Differences between adherent cells and suspended cells (CIKs) were no longer detectable. The third line in Figure 8 shows the results of an analysis carried out after 24 h of coculture and confirms these results. Adherent cells were DiD negative and positive for CD3 and CD25 and thus resemble the CIK phenotype.

## Discussion

We found that CIKs in coculture proliferated within 7 days with an average growth factor of 18.84, whereas controls proliferated with an average factor of 3.7 in the same period (Figure 1). The mechanisms responsible for this increase in proliferation of cCIKs have not yet been revealed. Four potential factors are discussed below: cell–cell interactions, humoral factors, MSC differentiation, and proliferation as a result of immune reactions against MSCs.

### Cell–cell Interactions vs. Humoral Factors

Many studies have reported MSC interactions with lymphocytes based on cell–cell contact. Various molecules on the surfaces of human MSCs, such as integrins, intercellular adhesion molecules (I-CAMs), or lymphocyte function-associated antigen-3 (LFA-3), have been found to bind to T-lymphocytes and mediate immunologic interactions [39]. Krampera et al. [40] for instance reported inhibition of proliferation and cytotoxicity of T-lymphocytes stimulated with their specific antigens when cocultured with MSCs. They further demonstrated that inhibition did not occur when MSC-conditioned medium was used instead of MSCs or when the experiments were performed in transwell membrane systems. Another group reported that MSCs can regulate the immune response of activated T-lymphocytes via the FAS ligand (FASL)/FAS-mediated pathway through cell–cell contact [55]. Vellasamy et al. [57] showed inhibition of PHA stimulated T-cells by MSCs in a dose dependent manner through cell–cell contact.

In contrast, other authors emphasize humoral aspects of interactions, showing for example, inhibition of proliferation of activated CD2+ lymphocytes by MSCs in a transwell membrane system [41]. Antibodies against hepatocyte growth factor and transforming growth factor β1 could abolish this effect, suggesting that these mediators may play a role in the observed effects [41]. Spaggiari et al. [34] described indoleamine 2,3-dioxygenase and prostaglandin E2-mediated suppression of proliferation, cytotoxicity, and cytokine production of NK cells. Maitra et al. [43] argued that MSCs do not constitutively secrete these soluble factors because culture supernatants from MSCs do not suppress lymphocyte proliferation, whereas cell-free supernatants from MSC-lymphocytes cocultures do.

Burr et al. [56] describe a more complex interplay between MSCs and lymphocytes, including cell contact, soluble mediators (prostaglandin E2 and transforming growth factor β), and indirect effects via manipulation of other antigen-presenting cells.

However, this study shows significantly increased proliferation of CIKs when cocultured with MSCs (p<0.05). As MSCs produce IL-7, IL-12, and IL-15 [42] and as these cytokines are known to stimulate the proliferation of lymphocytes and CIKs [7], [32], it seems supposable that the stimulating effects of the coculture are (at least) partly mediated by these (humoral) factors. But no significant influences on CIK proliferation were observed when CIKs were cultured in MSC-conditioned medium (p>0.1) (Figure 1), suggesting that the increased proliferation of cCIKs is mainly the result of cell contact-dependent mechanisms. However, only a few experiments with mediaCIKs have been performed owing to low numbers of MSCs. Further experiments are required to get more certain statements.

### Differentiation

Aside from cell–cell interactions and humoral factors, differentiation processes may also be responsible for the increased proliferation of CIKs in coculture. The disappearance of CD3−/CD25− cells from the coculture flasks alone may, for instance, support this theory. However, several arguments contradict this conclusion.

In contrast to pluripotent embryonic stem cells, CD34− multipotent mesenchymal stem cells have a more limited differentiation potential. They can differentiate into osteoblasts, chondrocytes, adipocytes, and other cell types but not into lymphocytes that originate from CD34+ haematopoietic stem cells (HSC) [27], [29]. However, some studies concerning murine MSCs argued that in contrast to human MSCs, murine MSC populations may include CD34+ cells [44]–[46].

More recent publications referring to very small embryonic-like stem cells (VSELs) should be considered as well. This small stem cell fraction is located in the bone marrow, and these cells express embryonic stem cell markers and can differentiate into cells of all three germ layers [47], [48]. If VSELs are present among MSCs, they might differentiate into CIKs and thus increase CIK numbers.

However, our results do not support differentiation theories, as shown by the results of our coculture experiments using DiD-labelled MSCs. In these experiments, DiD+ cells entered the coculture, and 4 h later, all cells were DiD−. At the same time, the phenotype switched from CD3−/CD25− to CD3+/CD25+, what is CIK phenotype. The differentiation of all MSCs into CD3+/CD25+ CIKs seems highly unlikely in such a short period. Even if this differentiation occurred, the cells should have stayed DiD+. In fact, they might have lost their DiD labelling as a result of membrane damage owing to CIK attacks for example, or thinning as a consequence of rapid proliferation, but the number of required coincidences for such an event to occur is substantial. On the other hand, 4 h seem to be an adequate time span for lysis. Furthermore, the occurrence of immune reactions is strongly consistent with the disappearance of DiD+ and CD3−/CD25− cells. Thus, lysis seems to offer a more plausible explanation.

### Immune Reactions

Overall, because the cells were derived from various allogeneic mice, the incidence of immune reactions seems very likely. Interestingly, MSCs are postulated to be non-immunogenic [30], [33]. In addition, many publications have even shown their suppressive and antiproliferative effects on T-lymphocytes and NK cells [17], [31], [32], [49]. These findings are very surprising with regard to the findings of the present study, wherein we show the proliferation-stimulating effects of MSCs on a cell population that combines elements of T-lymphocytes and NK cells. This raises questions about the possible mechanisms underlying these observed effects.

In fact, CIKs combine characteristics of both T-lymphocytes and NK cells, but they are neither. Furthermore, studies have shown that MSCs are not generally immunosuppressive and antiproliferative, but multiple factors and pre-conditions must be taken into account to explain their effects. Le Blanc [31], for example, reported that MSCs display dose-dependent inhibition of T-lymphocyte proliferation and that addition of low numbers of MSCs can even enhance lymphocyte proliferation. Spaggiari et al. [32] reported that allogeneic MSCs are lysed, not by resting NK cells but by IL-2-activated NK cells, which is interesting in the context of the results of the present study, as CIKs are also stimulated by IL-2. The same group showed that MSCs inhibit the proliferation of resting NK cells but only partially affect the proliferation of activated NK cells [34]. Eliopoulos and Fibbe [35], [38] have reported increasing CD8a+ T-lymphocytes, NK cells, and NKT cell numbers after coinfusion of CD34+ haematopoietic stem cells and allogeneic MSCs in the context of haematopoietic stem cell transplantation, which is contrary to the postulated immunosuppressive effects of MSCs and the related promising expectations of using MSCs in the therapy of GvHD. Above all, species-dependent distinctions must be taken into consideration because studies on MSCs concern predominantly human MSCs and remarkable differences exist among species [31], [50].

In summary, the immunologic interactions of MSCs are complex and poorly understood. According to current understanding, inhibitory as well as stimulatory effects occur side by side. Cell–cell interactions as well as humoral factors or immune reactions against MSCs are imaginable causes for the increased proliferation rates we observed in this study, whereas differentiation of MSCs to CIKs seems, according to existing data, unlikely.

### Effects on Phenotype, Cytotoxicity, and Vitality

For MSCs to be used to improve in vitro yield of CIKs, the possible impacts on the qualitative characteristics of cCIKs must be known. We found no evidence for any effects of MSCs on the phenotype of cCIKs (Figure 2). In addition, cytotoxicity against the analysed tumour cells (Wehi 164 S) did not seem to be compromised (Figure 4), which is of particular importance in terms of use in treating malignant diseases. Interestingly, coculturing seemed beneficial for vitality (Figure 3); however, further experiments are required to assess the significance of this possible benefit in more detail.

### Identification of MSCs

The identification of MSCs is not trivial for many reasons. Different extraction sites, inhomogeneous methods of isolation or cultivation, varying medium compositions, donor age, and many other factors [21], [23], [24] may lead to enrichment of different subsets of cells, which may also display disparities in differentiation potential, proliferation rate, or phenotype. Therefore, comparing MSCs, especially those of different species, often presents difficulties that are reflected in the ambiguity and even contradictions in the literature on MSCs. Specific markers or assays for the identification of MSCs, particularly for murine MSCs, still do not exist. To cope with this lack of tools, we identified the MSCs used in this study by criteria proposed by the ISCT for human MSCs but that in addition do consider publications about murine MSCs. These criteria include: adherence to plastic surfaces [25], [26], [27], [28]; spindle-shaped, fibroblast-like morphology [27], [54]; ability to grow in colonies and differentiate into osteoblasts in CFU-F-Assays [21], [28]; and phenotypical features [15], [28], [44]–[46], [51]–[53].

The criterion of adherence to plastic is a result of isolating MSCs in accordance to the protocol of Dobson [25], which is fundamentally based on the selection of plastic-adherent cells from bone marrow cells.

The spindle-shaped, fibroblast-like morphology is demonstrated in Figure 5A. As has been described by numerous authors [51], [54], a typical time-dependent transition through three morphologically distinct cell types could be observed when the cells were evaluated under light microscope: from thin spindle-shaped cells to wider spindle-shaped cells and to still wider spindle-shaped cells. The morphology is similar to that of fibroblasts, which is characteristic of MSCs.

Figure 5B shows the classical colony-growing shape of MSCs under normal as well as osteogenic differentiation conditions and the production of ALP when cultivated in osteogenic medium. The characteristic capability of MSCs to differentiate into osteoblasts in the presence of dexamethasone and ascorbic acid also supports the identification of adherent cells used in this study as MSCs.

Additionally, the identified MSCs were characterized phenotypically. In accordance with publications on murine MSCs, adherent cells were CD3−, CD31−, CD34−, CD44+, CD73+, and CD166+. Questions may arise concerning CD45 and CD90 because the results of this study show CD45+ as well as a CD45− population and a negative signal for CD90, which is contrary to the results of numerous authors who have postulated that MSCs have a CD45− and CD90+ phenotype [17], [28], [29]. This discrepancy in the literature seems to be due at least partly to differences between human and murine MSCs. For the most part, studies on MSCs have focussed on human MSCs, and these cells in fact are CD45− and CD90+. Even the ISCT criteria for MSCs include this CD45− and CD90+ phenotype, although these criteria are strictly for human MSCs [28]. Deeper review of the few and rare studies on murine MSCs reveals a more distinct understanding and heterogeneous phenotype. According to Phinney and colleagues [46], murine MSCs are a very heterogeneous cell population with many differences even between various mouse strains and may definitely contain CD45+ cells.

Baddoo and colleagues [45] describe murine MSCs as basically CD45− but state that plastic-adherent bone marrow cells are simply a mixture of fibroblastic and haematopoietic cell types. This group argues that isolation protocols relying only on plastic adherence of MSCs are insufficient for adequate separation of the various subsets of murine bone marrow cells. FACS analyses of these MSCs also revealed CD45+ and CD45− subsets, as demonstrated in our study. To obtain pure MSC cultures, an immune-depleting method is proposed instead by the group. In fact, by immune-depletion isolated murine MSCs did not express CD45 anymore. Just as well consistent with the findings of our study, neither the adherent MSCs nor the by immune-depletion isolated MSCs displayed CD90 on their surfaces. A possible explanation is delivered by Colter et al. [51], who reported that CD90 is not expressed on all murine MSCs and that its expression is dependent on the age and maturation state. Another important fact is that two different alloantigens occur for CD90: CD90.1 (Thy1.1) and CD90.2 (Thy1.2). The usual commercial antibodies bind either Thy1.1 or Thy1.2, depending on the respective clone. They usually do not cross-react, which makes analyses more complex to some extent. Overall, data about CD90 expression on murine MSCs are quite rare and inconsistent, and the picture becomes murkier when different mouse strains or extracting sites are considered. A study comparing MSCs isolated from bone marrow and those isolated from amniotic fluid found no Thy1.2+ MSCs in the latter and only 10% Thy1.2+ cells in the former [15]. Huiming and colleagues [52] analysed Thy1.1 on murine MSCs and detected no positive signals. This result was confirmed by another group comparing MSCs from different mouse strains with human MSCs; neither Thy1.1 nor Thy1.2 was found to be expressed on murine MSCs, but Thy1.1 was expressed on human MSCs [53].

Beyond these phenotypical considerations, human MSCs are known to possess enormous proliferation potential in vitro [29]. In contrast, murine MSCs reportedly have pure in vitro proliferation potential [45], [46], [53], which again agrees with the observations made while cultivating the MSCs for this study (not shown). However, the bone marrow cells used herein adhered to plastic surfaces, possessed spindle-shaped morphology, could proliferate in colonies and differentiate into osteoblasts in the presence of dexamethasone and ascorbic acid, and displayed a CD3−, CD31−, CD34− and CD44+, CD73+, CD166+ phenotype; all these features match to a large extent with the characteristics of murine MSCs [44]–[46], [51]–[53].

## Conclusions

CIKs are a promising new tool for anti-tumour therapy. Because CIKs are quite rare in vivo, many groups have tried to optimize their in vitro culture yield. This study investigated the influence of MSCs on the proliferation, phenotype, vitality, and cytotoxicity of CIK cells. Increased proliferation was demonstrated, and higher vitalities were measured. No impacts on phenotype or cytotoxicity were revealed. The increased proliferation seems to be attributable primarily to interactions resulting from cell–cell contact. A subordinate influence of humoral factors seems supposable, but until now - perhaps because of a small number of experiments - could not be ascertained with statistical significance. Because phenotypical MSCs were no longer detectable after 4 h of coculture, immune reactions of CIKs against MSCs appear to be of importance as well. Experiments using DiD-labelled MSCs suggested that differentiation processes were not responsible for the observed effects.
